# Myotube Formation and Cellular Fusion Are Diminished Due to Low Birth Weight in Piglets

**DOI:** 10.3390/ijms26072847

**Published:** 2025-03-21

**Authors:** Katja Stange, Monika Röntgen

**Affiliations:** Working Group Cell Biology of Muscle Growth, Research Institute for Farm Animal Biology (FBN), 18196 Dummerstorf, Germany; stange.katja@fbn-dummerstorf.de

**Keywords:** myogenic differentiation, low birth weight, myofiber fusion

## Abstract

Low birth weight (LBW) in various species leads to a pronounced skeletal muscle phenotype and can serve as a model to study muscle formation and draw conclusions for normal and pathological development. We aimed to elucidate in detail how the differentiation of muscular stem cells and their progeny are disturbed in piglets born with LBW. We isolated primary muscle cells from LBW piglets and their normal siblings with two different approaches: (1) single cells from two functionally divergent subpopulations (previously named “fast” and “slow”) and (2) cells derived from isolated, intact myofibers. Subsequently, we analyzed their proliferative and differentiative capacity by determining proliferation rate, migration behavior, myotube formation, and myogenic gene and protein expression. LBW led to a decreased proliferation rate and migration potential in cells from the subpopulation fast group. Cells from LBW piglets were generally able to differentiate, but they formed smaller myotubes with less incorporated nuclei, leading to a diminished fusion rate. Myogenic gene expression was also significantly altered due to pig birth weight. Overall, early postnatal muscle development in LBW was disturbed at several crucial steps involving the establishment of a reserve stem cell pool, movement of cells towards existing myofibers, and the ability to form nascent myofibers.

## 1. Introduction

Intrauterine growth retardation and low birth weight (LBW) can lead to a pronounced skeletal muscle phenotype, which can cause life-long impairments in muscle growth, e.g., in sheep, cattle, and pig [1,2,3]. The incidence of a muscle phenotype is higher in polytocous species like the pig, where litter size and birth weight variability have been greatly enhanced due to breeding programs [4,5]. This leads to a higher number of LBW piglets born [4,6,7], which is associated with higher morbidity and mortality [8], developmental disadvantages, increased infection risk [1,9,10], behavioral problems [9,11,12], and a worse growth rate and meat quality [1,4]. In vivo, the muscular phenotype in LBW pigs is already seen at birth and is mainly characterized by a reduced muscle tissue mass related to body weight and less myofibers, showing that prenatal myogenesis (formation of new myofibers) is impaired [1,13].

In the pig, myogenesis occurs in two distinct waves and is finished at about day 90–95 of gestation [14]. Embryonic myoblasts form primary myofibers between day 35 and day 60 of gestation. Thereafter (day 55–90 of gestation), fetal myoblasts generate secondary fibers around them [1,14]. The main component of pre- and postnatal myofiber development is the fast accumulation of myofibrils consisting largely of Myosin. Different Myosin isoforms are found in skeletal muscle tissue, determining distinct muscle fiber types (slow, fast or hybrid) [15]. In contrast to other species, all adult fast Myosin isoforms (MyH2 > MyH1 > MyH4 coding for MyHC 2A, MyHC 2X, and MyHC 2B) are prenatally expressed in the pig from about day 35 of gestation on. Nevertheless, slow MyH7 (coding for MyHC1) and fast embryonic MyH3 (coding for MyHC emb) are the dominating isoforms until day 77 of gestation. Starting toward the end of gestation, an increased expression of all fast adult Myosin isoforms occurs, with MyH1 showing a dramatic increase, followed by MyH2 and MyH4 [16]. In addition, maturation of pig skeletal muscle includes a conversion of secondary type II to type I fibers that already commence at late gestation and continue until week 3–8 postpartum [17,18].

It has been shown that a variety of factors and insults (like high ovulation rate, uterine crowding, placenta inefficiency associated with nutrient, and oxygen limitation) have adverse effects on pig myogenesis, specifically on secondary fiber number and growth [1,9,13,19].

Reduced protein synthesis, increased proteolysis, and reduced muscle tissue capillarization contribute to reduced muscle growth and slower myofiber maturation [13,20]. The authors of [1] concluded that prenatal cell proliferation, differentiation, and protein accretion are distinctly below average in LBW piglets. However, most data come from studies investigating changes in muscle tissue, whereas investigations using primary muscle cells to explore the muscular phenotype of LBW piglets are quite rare. It was reported that muscle cell abundance and characteristics are changed due to LBW in pigs [20,21,22]. Of the various cell types (hematopoietic cells, microvascular cells, fibro/adipogenic cells, and fibroblasts) found in muscle, satellite cells (SC) are the most critical, as postnatal muscle growth, maintenance, and regeneration greatly rely on them [8,23,24,25]. SC become quiescent in adult muscle and have the ability to self-renew and settle in their stem cell niche [26,27,28,29]. However, during postnatal myogenesis, a majority of SC is activated (in pig about 90% during the first week of life) and give rise to proliferating myoblasts [30]. The following processes of lineage commitment and myogenic differentiation are directed by a series of regulators and transcription factors [31]. After several rounds of proliferation, myoblasts exit into differentiation and express Myogenin (MyoG), a marker of the initiation of differentiation [29,30]. Myogenic cells subsequently fuse into multinucleated myotubes [32].

The development and growth of muscles require the terminal differentiation of myogenic precursor cells into myotubes/myofibers [33]. Cells that are unable to differentiate terminally undergo apoptosis, a physiological process also seen in the development, maintenance, and regeneration of many tissues [34]. During myogenesis, apoptosis is thought to eliminate excess myoblasts, and it is estimated that up to 30% of myoblasts die due to programmed cell death in in vitro differentiation assays [35,36]. Apoptosis is enhanced by stress conditions such as oxidative stress or mitochondrial dysfunction and leads to pathological changes like muscular atrophy [34,35].

Myotubes can form via two different mechanisms: (1) myoblast–myoblast fusion to form new/nascent myotubes (primary fusion) or (2) myoblast–myotube fusion for the growth and hypertrophy of existing myotubes (secondary fusion) [37]. Myomaker (Mymk) was the first identified muscle-specific fusion factor. The loss of Mymk in mice results in the presence of mononucleated Myosin+ cells, which fail to fuse. During myoblast fusion, cell adhesion proteins aggregate at the site of membrane fusion; one of these is Myoferlin (MyoF) [37,38,39]. It was found in mononuclear cells during proliferation and early differentiation, and is downregulated when cells mature [39,40]. MyoF is crucial for myoblast–myoblast and myoblast–myotube fusion but also for myotube fusion as it is expressed at the contact site between two myotubes [38,39]. The Mannose receptor type I (Mrc1) is a transmembrane protein required for myoblast–myotube fusion. Mrc1-null myotubes remain small and contain less nuclei because myoblast nuclei cannot fuse into the nascent myotubes during their growth stage [41].

In the pig, myofiber number is fixed at birth, and the early postnatal growth of existing fibers depends on the sustained expansion and myonuclei accretion of SC. SC subpopulations expressing myogenic markers Pax7 and/or Myf5 accrue already during fetal development and contribute to the growth of secondary muscle fibers in utero [3,25]. A part of those Pax7+ SC pass into the fetal period, become enveloped by the basal lamina of developing myofibers, and establish the postnatal SC pool [25,42]. Thus, it seems possible that prenatal conditions leading to LBW will have negative effects on peri- and postnatal SC functionality, specifically on their ability to differentiate.

Therefore, we used two different in vitro approaches: cellular subpopulations (SPF and SPS) and cells derived from single, intact myofibers to investigate the differentiation capacity of MPC isolated from 4–5-day-old piglets with NBW and LBW in detail. In general, muscle (stem) cells and their progeny are a very heterogeneous population, and subpopulations showing differences in proliferation, adhesion, fusion ability, migration/response to growth factors, or self-renewal have been identified in various mammalian and avian species [43,44,45,46,47,48,49,50,51,52,53,54]. We identified two myogenic subpopulations named subpopulation fast (SPF) and subpopulation slow (SPS) according to their proliferation and adhesion potential, which we characterized in detail [20,55,56]. The SPF consists mainly of committed MPC and cells marked for terminal differentiation (high expression of *Myf5* and *MyoG*). In accordance with that, they achieve higher fusion rates and form larger myotubes that SPS cells. In contrast, SPS cells are characterized by a higher number of Pax7+ cells (showing a more immature phenotype) and a lower differentiation potential resembling properties of a reserve cell population. Using SPF and SPS cells instead of mixed mass cultures, allowed us to consider the very different functions of these populations in postnatal myogenesis.

In addition, we used the intact myofiber model because myogenic cells firstly remain in their native position at the myofiber, and their properties are more similar to muscle tissue than in standard cell culture [29,57,58]. However, the isolation of single myofibers and the emerging cells is rarely used in larger vertebrates. In a previous study, we described the isolation and cultivation of intact, single myofibers from the porcine fibularis tertius muscle [59]. Cells could either proliferate on the myofiber or adhere to cell culture dishes in order to differentiate into myotubes. Their isolation as well as the myogenic differentiation of the cells were never performed in LBW piglets before.

## 2. Results

**Figure 1:**
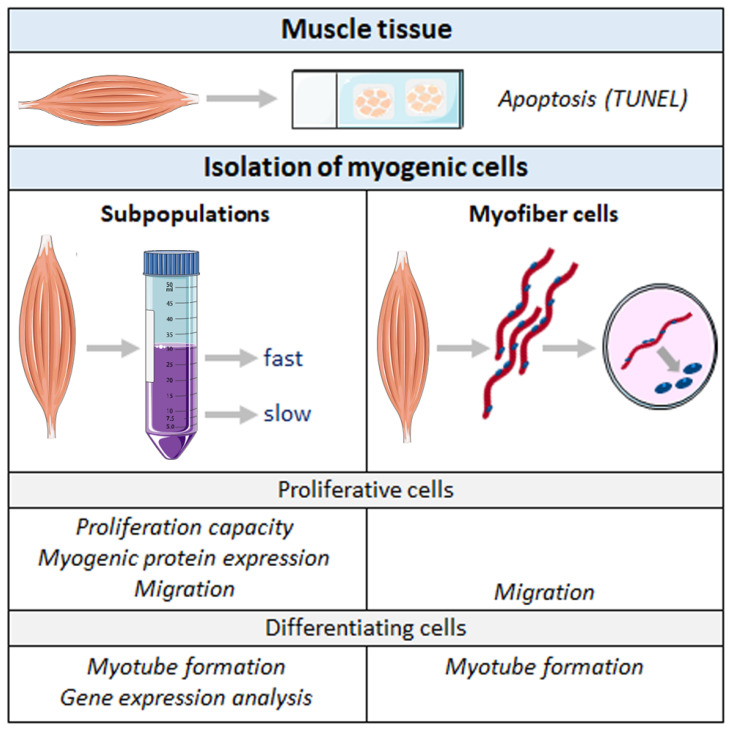
Overview of experiments and sample material. Cryosections were used to identify apoptotic cells in the longissimus dorsi (LD) muscle. Proliferation rate, expression of myogenic markers MyoG and MHC, and cell migration were assessed in proliferative cell subpopulations. Gene expression, MyoG protein expression, and different parameters of myotube formation were analyzed during the myogenic differentiation of cellular subpopulations. Myofiber-derived cells were used to study migration (proliferative cells) and myotube formation (differentiating cells). Samples from NBW and LBW piglets were compared for each parameter. Copyright schematic items (e.g., muscle, falcon tube): 2006, Les Laboratories Servier.

An overview of the conducted experiments and respective sample material can be found in Figure 1. We investigated the proliferation rate, myogenic protein expression, and cell migration in proliferative cells. Furthermore, the differentiation capacity was studied in detail by assessing myotube formation, MyoG and Myosin protein expression, and the expression of a variety of myogenic genes in differentiating cells. Analyses were complemented by using cells derived from isolated and cultivated single myofibers [59] and by investigating their migration and myotube formation.

### 2.1. Growth and Myogenic Marker Expression of Cell Subpopulations from NBW and LBW Piglets During Proliferation

**Figure 2:**
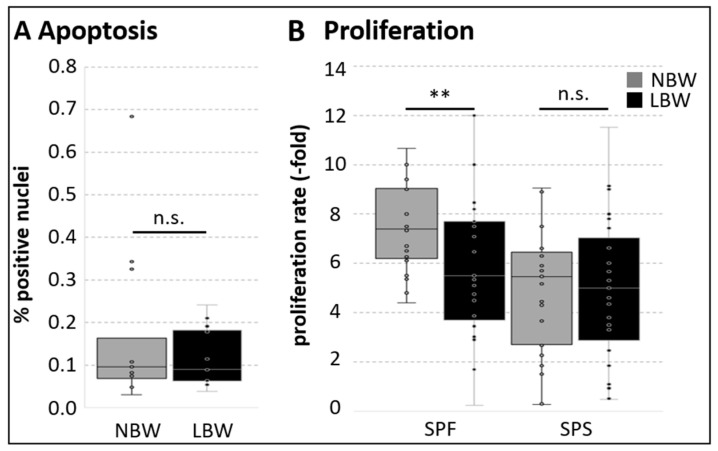
Apoptosis and proliferation in muscle (-derived cells). (**A**) Apoptotic cells in cryosections of LD muscle from NBW and LBW piglets were detected via TUNEL assay. Apoptosis levels were generally low, and no significant difference was detected (*n* = 12). The Mann–Whitney Rank Sum Test was used for statistical analysis. (**B**) The proliferation of muscle cell subpopulations was determined at day 8 after isolation after 4 days of cell growth (passage 1, *n* = 22). In SPF, the proliferation rate was significantly lower in cells obtained from LBW piglets, whereas no difference was seen in SPS. One tailed student’s *t*-test was used for statistical analysis (comparing NBW and LBW within each subpopulation), ** *p* ≤ 0.01, n.s. not significant.

We first quantified the number of apoptotic cells using the common TUNEL assay to detect DNA breaks (Figure 2A). Less than 0.2% of cells in muscle tissue were found to be positive, and no difference depending on birth weight was detected. The proliferation rate was assessed during passage 1 (Figure 2B). Equal cell densities were seeded at day 4 after isolation, and cell number was determined 4 days later. The proliferation rate within SPF was significantly lower in cells derived from LBW piglets (6.02 ± 3.04 fold vs. 7.48 ± 1.8 fold). In SPS cells, which showed a lower proliferation rate, no difference depending on birth weight was detected.

Cell viability and size were continuously assessed during experiments at day 0, 4, and 8 after isolation. Cell viability amounted to 90.32% ± 9.61% throughout cultivation (*n* = 30), and mean cell size was 10.9 µm ± 2.89 µm (*n* = 30).

**Figure 3:**
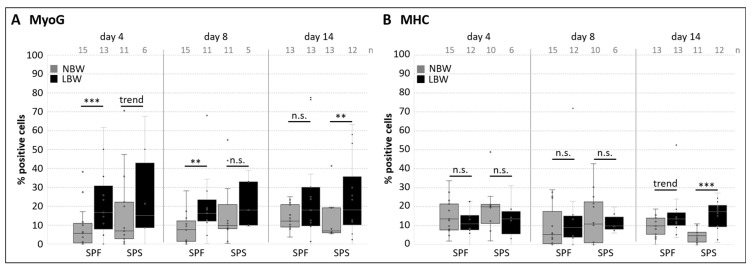
Myogenic marker expression in isolated subpopulations. Protein expression of MyoG and MHC isoforms was detected using flow cytometry analysis. The number of biological replicates (*n*) is given in the upper part. One-tailed student’s *t*-test was used for statistical analysis (comparing NBW and LBW within each subpopulation at each respective day of analysis), *** *p* ≤ 0.001, ** *p* ≤ 0.01, trend *p* ≤ 0.1, n.s. not significant. (**A**) MyoG expression in SPF and SPS was generally elevated in LBW piglets. (**B**) At day 14 after isolation, a higher proportion of cells from both subpopulations expressed Myosin HC isoforms in LBW piglets.

Next, the protein expressions of MyoG and Myosin heavy chain isoforms were determined under conditions promoting proliferation (Figure 3). Both are crucial for myogenic differentiation, and a considerable number of cells already expressed these proteins. Interestingly, a higher percentage of cells from LBW piglets expressed MyoG (Figure 3A). The effect was statistically significant or showed a trend at day 4 and 8 in SPF and at day 4 and 14 in SPS. For MHC, only at the latest selected time point (day 14 after isolation) a significant difference between NBW and LBW was observed, with a higher number of MHC+ cells in the SPS population of LBW piglets (Figure 3B).

### 2.2. Migration of Cell Subpopulations and Myofiber-Derived Cells from NBW and LBW Piglets During Proliferation

**Figure 4:**
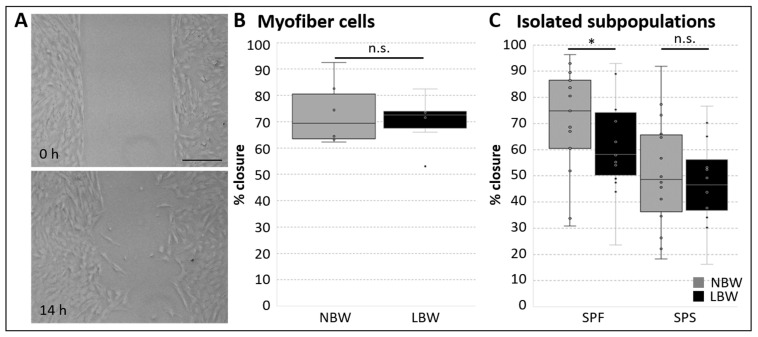
Migration behavior of myogenic cells. Cell subpopulations or myofiber-derived cells were seeded in exclusion zone assay, and migration was assessed after 19–20 h or 14 h, respectively. One-tailed student’s *t*-test was used for statistical analysis (comparing NBW and LBW (within each subpopulation)), * *p* ≤ 0.05, n.s. not significant. (**A**) Exemplary image of migration assay right after barrier removal (0 h) and 14 h later. The width of the cell-free gap/barrier is 500 µm ± 50 µm (ibidi); scale bar 200 µm. (**B**) Using cells obtained from single myofibers, no difference in migration depending on birth weight was detected (*n* = 6). (**C**) SPF cells from LBW piglets migrated significantly slower than those from NBW piglets, whereas no difference was detected in SPS cells (*n* = 12–14).

In order to assess cell migration capacity, we used the barrier/exclusion zone assay [60,61] and monitored the closure of the gap after a certain time via microscopy (Figure 4A), and the size of the remaining gap was calculated. To ensure that the cell-free region was occupied by migrating cells and that results were not influenced by cell proliferation, cells were treated with the cell cycle antagonist Mitomycin C (MMC) before removal of the barrier [13,62]. Both myofiber-derived cells and cell subpopulations were able to migrate into the cell-free region and mostly closed the gap in less than 1 day. For cells obtained from single myofibers, no significant difference between NBW and LBW piglets regarding migration speed was observed (Figure 4B). Only the use of distinct cell subpopulations revealed a difference depending on birth weight, showing slower migration (decreased closure) in SPF cells from LBW piglets (Figure 4C). SPS cells migrated slower in general without any difference between NBW and LBW.

### 2.3. Differentiation Capacity of Muscle-Derived Cell Subpopulations

**Figure 5:**
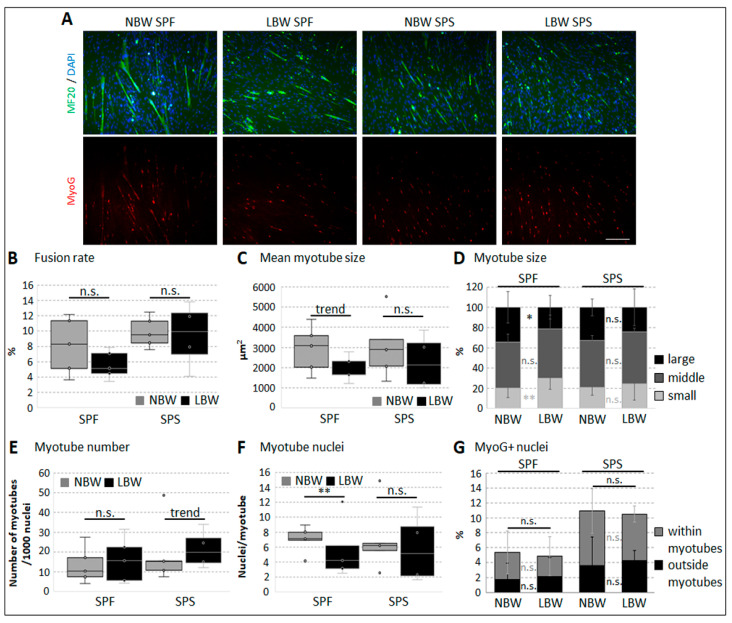
Myotube formation in isolated subpopulations. Cells from SPF and SPS obtained from NBW or LBW piglets were allowed to differentiate, and antibody-based immunofluorescence staining was conducted after approximately 1 week of differentiation. (**A**) Representative images of differentiated cells, which were simultaneously stained against MHC (MF20, green), MyoG (red), and DAPI (blue). Multinuclear myotubes and MyoG+ nuclei can be clearly seen. Scale bar 200 µm. (**B**–**F**) Quantitative analyses of cell cultures stained against MHC and DAPI. One-tailed student’s *t*-test was used for statistical analyses comparing NBW and LBW within each subpopulation (*n* = 5). (**B**) Fusion rate (percentage of nuclei within myotube area) did not differ within subpopulations depending on birth weight. (**C**) The mean size of identified myotubes tended to be smaller in SPF cells from LBW piglets. (**D**) For the detailed assessment of myotube size, they were categorized into three groups according to quartiles of frequency distribution (25% small, 50% middle, and 25% large). In SPF cells from LBW piglets, significantly less large myotubes and more small myotubes were found. (**E**) The number of myotubes formed per 1000 cell nuclei (irrespective of their size) tended to be higher in SPS cells from LBW piglets compared to NBW. (**F**) The mean number of nuclei found within each myotube was significantly lower in SPF cells of LBW piglets. (**G**) The percentage amount of MyoG+ nuclei did not differ between cells from NBW and LBW piglets in both subpopulations. The proportion of MyoG+ nuclei found within or outside of myotubes showed no differences depending on birth weight as well. One-tailed student’s *t*-test was used for statistical analyses comparing NBW and LBW within each subpopulation (*n* = 3), ** *p* ≤ 0.01, * *p* ≤ 0.05, trend *p* ≤ 0.1, n.s. not significant.

Direct cell–cell contact, serum starvation, and the presence of Matrigel successfully induced myogenic differentiation in porcine myogenic cell subpopulations, as shown by MyoG expression and the formation of multinucleated, MHC-positive myotubes (Figure 5A). MyoG+ nuclei were found outside of and in myotubes (Figure 5G). In general, the fusion rate and the number of myotubes did not differ between cells isolated from NBW or LBW piglets (Figure 5B,E). Nevertheless, myotubes formed by cells in SPF were significantly smaller (Figure 5C,D), and thus contained less myonuclei (Figure 5F), if they were isolated from LBW piglets.

Next, we assessed the expression of selected target genes being important during different stages of myogenic differentiation. The earlier myogenic markers *Pax7*, *Myf5*, *MyoD1*, and *MyoG* were present in all samples during the differentiation process. Cells in SPS from LBW piglets expressed significantly less *Pax7* and *MyoG* when inducing differentiation and significantly more *Myf5* at day 4 of differentiation compared to NBW.

**Figure 6:**
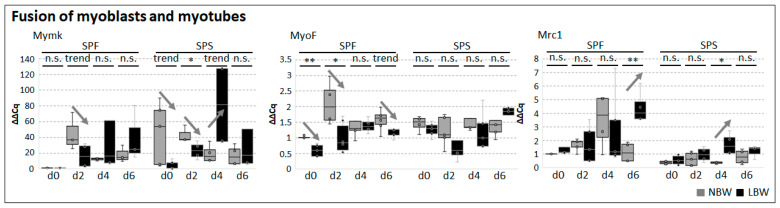
Expression of genes responsible for myoblast/myotube fusion in isolated myogenic subpopulations. The expression of *Mymk*, *MyoF*, and *Mrc1* was assessed in differentiating cells via qRT-PCR. Arrows highlight significant differences or trends and their direction (up- or downregulation) between cells derived from NBW and LBW piglets. One-tailed student’s *t*-test was used for statistical analyses comparing NBW and LBW within each subpopulation at each respective day of analysis (*n* = 6), ** *p* ≤ 0.01, * *p* ≤ 0.05, trend *p* ≤ 0.1, n.s. not significant.

**Figure 7:**
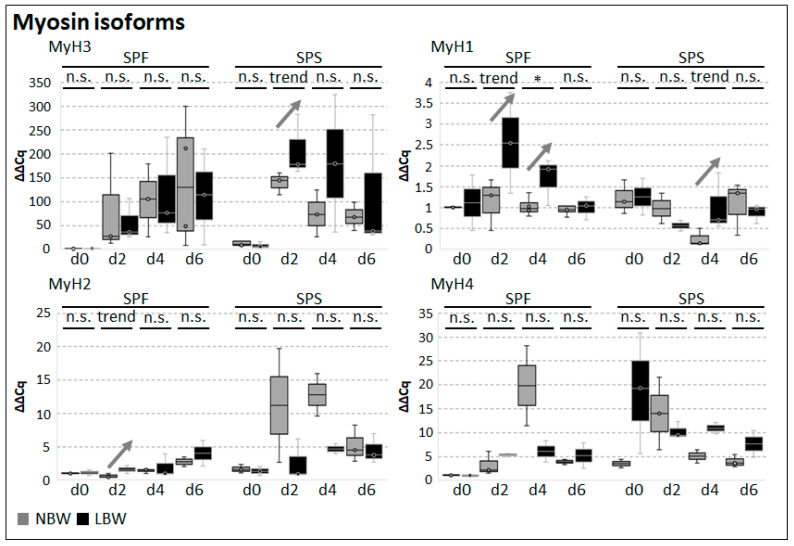
Gene expression analysis of MyH isoforms in isolated subpopulations. The expression of the Myosin isoforms *MyH3* (eMyH), *MyH1*, *MyH2*, and *MyH4* was assessed in differentiating cells via qRT-PCR. Arrows highlight significant differences or trends and their direction (up- or downregulation) between cells derived from NBW and LBW piglets. One-tailed student’s *t*-test was used for statistical analyses comparing NBW and LBW within each subpopulation at each respective day of analysis (*n* = 4–5), * *p* ≤ 0.05, trend *p* ≤ 0.1, n.s. not significant.

However, our analyses concentrated on genes important for later stages of differentiation: *Mymk*, *MyoF*, *Mrc1* (Figure 6), and the Myosin isoforms *MyH3* (MyHC emb), *MyH1*, *MyH2*, and *MyH4*, encoding for the proteins Myosin 3, Myosin 2X, Myosin 2A, and Myosin 2B, respectively (Figure 7). For *Mymk*, the highest expression level in NBW was reached at day 2 (SPF) and day 0 (SPS); in LBW, the maximum was only reached at day 6 (SPF) and day 4 (SPS). *Mymk* expression was statistically lower or tended to be lower in cells from LBW piglets compared to NBW (SPF day 2, SPS day 0 and 2). Contrastingly, at day 4 after induction, *Mymk* expression tended to be higher in SPS from LBW piglets. *MyoF* also showed reduced expression in SPF cells of LBW piglets (day 0, 2, and 6). The expression level of *Mrc1* in SPS was rather low and constant in comparison to SPF. The expression was significantly enhanced in cells from SPF (day 6) and SPS (day 4) of LBW piglets compared to NBW.

All tested Myosin gene products showed a low expression when differentiation was induced, whereas the majority was upregulated during the process (Figure 7). *MyH3* and *MyH2* tended to be higher at day 2 in SPS or SPF from LBW piglets, respectively, but otherwise, their expression was not significantly different between cells from NBW and LBW piglets. For *MyH1* expression, in cells from LBW piglets, an upregulation was observed at day 2 (SPS) and 4 (both subpopulations) compared to NBW piglets. *MyH4* expression showed no significant differences between cells derived from NBW and LBW piglets at all tested time points.

### 2.4. Differentiation Capacity of Myofiber-Derived Cells

**Figure 8:**
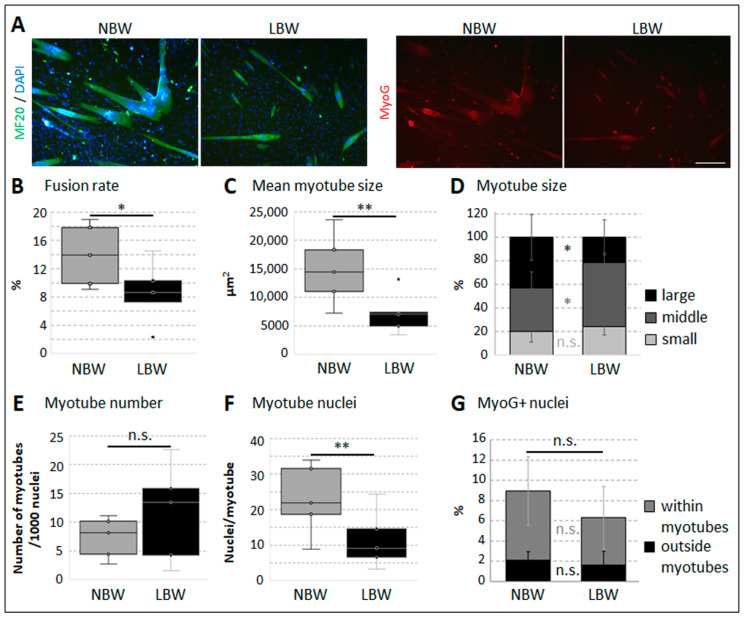
Myotube formation in myofiber-derived cells. Myogenic cells derived from single myofibers isolated from NBW or LBW piglets were allowed to differentiate, and antibody-based immunofluorescence staining was conducted after approximately 1 week of differentiation. One-tailed student’s *t*-test was used for statistical analyses comparing NBW and LBW (*n* = 5), ** *p* ≤ 0.01, * *p* ≤ 0.05, n.s. not significant. (**A**) Representative images of differentiated cells simultaneously stained against MHC (MF20, green), MyoG (red), and DAPI (blue). Multinuclear myotubes and MyoG+ nuclei can be clearly seen. Scale bar 200 µm. (**B**–**F**) Quantitative analyses of cell cultures stained against MHC and DAPI. (**B**) Fusion rate was significantly lower in cells derived from LBW piglets. (**C**) The mean size of identified myotubes was significantly lower in cells from LBW piglets, as well. (**D**) Myotubes were categorized into three groups according to quartiles of frequency distribution of their size (25% small, 50% middle, and 25% large). In cells from LBW piglets, significantly less large myotubes and, consequently, more middle-sized myotubes were found. (**E**) The number of myotubes formed per 1000 cell nuclei (irrespective of their size) did not differ depending on birth weight. (**F**) The mean number of nuclei found within each myotube was significantly lower in cells of LBW piglets. (**G**) The percentage of MyoG+ nuclei as well as the proportion of MyoG+ nuclei found within or outside of myotubes did not differ between cells from NBW and LBW piglets.

Myofiber-derived cells were obtained with a fundamentally different method than the isolated subpopulations SPF and SPS. Thus, the use of these cells can give further insight, and we performed the myogenic differentiation assay with myofiber-derived cells, as well. Again, the formation of multinucleated myotubes and numerous MyoG+ cells was observed due to cell–cell contact, serum starvation, and Matrigel (Figure 8A). The fusion rate of cells derived from myofibers from LBW piglets was significantly lower compared to NBW piglets (Figure 8B). The mean myotube size (Figure 8C), as well as the proportion of large myotubes (Figure 8D), was also reduced due to LBW, and consequently, less cell nuclei were present in each myotube (Figure 8F). Interestingly, the myotube number was not affected by birth weight in vitro (Figure 8E). The proportion of cells being positive for MyoG as well as their localization within or outside of myotubes did not differ between cells from NBW and LBW piglets (Figure 8G).

## 3. Discussion

The pig is a highly useful biomedical model for understanding muscle development and the behavior of myogenic progenitor cells. In the present study, we compared the in vitro differentiation capacity of myogenic cells from LBW piglets to cells obtained from NBW piglets and included further experiments to elucidate possible reasons for birth weight-associated differences. In contrast to other studies, we used no mass cultures of satellite cells but two functionally distinct subpopulations isolated from digested muscle tissue [55] and a more complex model, in which myogenic cells actively migrate from single isolated muscle fibers. We would like to emphasize that the obtained cells/cell populations differ from each other and therefore have specific advantages. Cells obtained from intact muscle fibers are thought to reflect in a better way the physiological situation, whereas cells isolated from muscle tissue show the intrinsic functional capacity of the cells [56,63,64].

The vast majority of SPF and SPS cells exhibits a myogenic phenotype, as shown by expression of the myogenic markers Desmin and MyoG [55]. Furthermore, cells from SPF are highly committed (enhanced *Myf5* expression right after isolation) and show a higher capacity to differentiate into myotubes combined with a lower percentage of Pax7+ cells during proliferation [55,56]. In our previous studies, functional properties and the physiological role of cells from SPF and SPS were investigated in NBW piglets. However, no functional data are available for SPF and SPS cells derived from LBW piglets, although there is a high risk that they are negatively affected due to less favorable prenatal conditions. The isolation and cultivation of single, intact myofibers, used to complement our analyses, were not performed previously with muscles from LBW piglets. Thus, migration behavior and differentiation capacity (myotube formation) of porcine myofiber-derived cells were examined for the first time. To enable assumptions about the in vivo situation during muscle development and growth from assays conducted in vitro, one has to take into account the physiological situation in the animal, e.g., the bioavailability of cells. In muscle sections from LBW piglets, the number of myogenic cell nuclei within muscle fibers is significantly reduced [1,4,20,65]. Nissen and colleagues [21] also stated that the number of viable cells in myogenic cultures from low weaning weight piglets was lower. In accord with that, we could isolate significantly less cells per gram muscle from LBW muscle compared to their NBW littermates, which points to a reduced SC pool and thus lower provision of myonuclei and DNA for hypertrophic growth [20].

In cross-sections of muscle tissue, we found 0.4 nuclei per myofiber in 4-day-old piglets [20], which increased to 1.6–1.8 nuclei per myofiber in 20-week-old pigs [66]. In single isolated myofibers, we found approximately 34 cell nuclei per mm myofiber directly after isolation from 4-day-old piglets [59]. These myofiber-derived cells probably represent a mixed population of cells, and we hypothesized that both cellular subpopulations, SPF and SPS, were included in their physiological proportions at the time point of myofiber isolation. When isolated as single cells from muscle tissue, a ratio of 35% (NBW)/42% (LBW) was found in the SPF and of 65% (NBW)/58% (LBW) in the SPS [20]. We assume that the SPS, as when using gradient centrifugation, constitutes the majority of cells, but again represented a smaller proportion of all cells in LBW piglets than in NBW piglets. Nevertheless, we cannot test for the homogenous migration of cells from isolated fibers and subsequent adherence to the cell culture dish; therefore, the exact proportion of SPS or SPF cells migrating from the fiber remains unclear. Since SPF cells seemed to show a higher migration capacity, it can still be presumed that these cells predominantly leave the myofibers and mainly adhere to the dish. Later on, these mixed cell populations were cultivated together in this experimental setup, and their proportions and characteristics might change as they affect each other in co-culture [56].

Although programmed cell death is a physiological process to ensure tissue development, growth, and homeostasis [67,68], an elevated level in muscle tissue can also be caused by disturbances in differentiation, as found in piglets suffering from congenital splay leg syndrome [69]. Nevertheless, we saw that LBW does not lead to enhanced apoptosis in muscle sections and is, therefore, not causative for a lower cell number. Consequently, our results showed that the MPC pool in LBW piglets is not prematurely exhausted due to cell death at the investigated time point. Nevertheless, we cannot exclude increased apoptotic events to occur in the prenatal period. Still, a considerably smaller number of MPC seems to exist in muscle due to LBW right after birth. This argues for an a priori smaller muscle stem cell pool in LBW piglets, as we hypothesize that SPF cells arise from the pool of SPS [55]. Thus, this process might be reduced if the stem cell pool is diminished.

When using mass mixed cultures, no (or only subtle) changes in the proliferation of cells isolated from LBW muscle could be detected [21,70]. Since we hypothesized that this system levels out birth-dependent differences, we did not focus on the proliferation of myofiber-derived cells, because we found a mixture of both subpopulations, as well. Indeed, when analyzing cells from SPS and SPF separately, otherwise masked differences could be elucidated. The proliferative capacity of cells in SPS was independent of birth weight, whereas the ability to proliferate was significantly reduced in cells derived from the SPF of LBW piglets. A higher level of the differentiation marker MyoG at early time points of cultivation might contribute to decreased proliferation, since MyoG is known to be expressed in myocytes but not in (proliferating) myoblasts [71]. It should be noted that proliferation assays started with similar seeding densities and still showed slower proliferation in cells from LBW. In vivo, this deficit might add up to the general lower number of MPC being available.

A lower differentiation capacity in myogenic cells from low birth weight or low weaning weight pigs was seen in previous studies, but due to the use of mixed mass cultures, in-depth details could not be determined [21,70], for instance regarding the distinct roles of cellular subpopulations in myofiber formation. The consistent proliferative activity of SPS cells from LBW piglets can be interpreted in a way that a normal reserve cell pool (consisting of cells from SPS) is maintained in LBW. In addition, SPS cells are hypothesized to play a role in myofiber formation (cell–cell fusion) and myofiber prolongation rather than in postnatal hypertrophic myofiber growth (cell–myofiber fusion). This is consistent with observations in avian species, where the slowly proliferating MPC subpopulation is preferably fusing to form new myofibers [32]. Cell migration is comparable in SPS from LBW compared to NBW, and slightly more myotubes could be formed (trend). The fusion rate, as well as the size of myotubes and the number of nuclei per myotube, could compete with cells from NBW siblings, and thus, main parameters of myogenic differentiation are held constant in SPS despite of a LBW phenotype.

Contrarily, cells of the SPF have an important role in providing myonuclei for hypertrophic myofiber growth [55]. The reduced proliferation rate of these cells can lead to negative effects on myofiber development. Interestingly, in myofiber-derived cells, migration rate and myotube number remained unchanged, probably due to the higher proportion of cells from SPS cells in the mixed population. However, other main parameters clearly confirmed the deficits of SPF cells in hypertrophic growth in LBW. Indeed, the proportion of large myotubes was lower with cells from SPF in LBW piglets and formed myotubes had fewer myonuclei as well. Importantly, similar results (but in an even higher intensity) were found when myofiber-derived cells were used. However, in addition to smaller myofibers with lower myonuclei, investigation of myofiber-derived cells clearly revealed a reduced fusion rate in cells from LBW piglets. This coincides with a previously found decreased myofiber cross-sectional area in the muscle tissue of LBW piglets in vivo [20] and might contribute to a reduction of muscle mass in vivo.

In accord, genes involved in the regulation of fusion processes, specifically *Mymk* and *MyoF*, were downregulated in isolated cells from LBW piglets. *Mymk* expression, needed for myoblast fusion and therefore the formation of nascent myotubes by SPS cells [31,72], was reduced at early time points in SPS. Nevertheless, expression only peaked at day 4 in LBW (4 days later than in NBW) and might point to a different dynamic that prevents a severe effect on in vitro differentiation in this subpopulation. *MyoF* expression was clearly affected in SPF cells of LBW piglets in early as well as in later time points of differentiation, correlating with the reduced migration capacity of this cell population towards existing myofibers. This effect might be combined with a reduced attraction of these cells by signals secreted from myofibers, further intensifying the inability to incorporate myonuclei into nascent myofibers. An increased expression of *Mrc1* at comparably late time points in LBW in both subpopulations cannot compensate for the disturbances in myogenic differentiation in LBW. Despite these impairments during myogenic differentiation, crucial Myosin gene products could be detected, including adult Myosin isoforms, although gene expression must not be reflective of protein expression. Nevertheless, a higher *MyH3* (embryonic) expression in the SPS reserve cell pool in LBW might argue for a delay in switching from embryonic to adult forms.

Although in vitro assays are not able to fully reconstruct the large variety of processes in muscle development in vivo (e.g., multidimensional myotube alignment or muscle fiber maturation), they still provide valuable insights into the myogenic differentiation capacity of muscle progenitor cells, which is clearly diminished due to LBW in pigs.

## 4. Materials and Methods

### 4.1. Animals and Muscle Dissection

Animals were housed, selected, and slaughtered as described previously [20]. In brief, 4–5-day old female German Landrace piglets were provided from the experimental pig unit of the Research Institute for Farm Animal Biology and slaughtered according to the guidelines of the Animal Care Committee of the State Mecklenburg-Western Pomerania, Germany, based on the German Law of Animal Protection. No animal experiment was conducted, since animals were not manipulated before slaughter. In total, 47 piglets with normal birth weight (NBW, 1.38 kg ± 0.12, ranging from 1.2 to 1.7 kg) and 46 piglets with low birth weight (LBW, 0.91 kg ± 0.11, ranging from 0.7 to 1.2 kg) from 48 litters were used for the presented experiments. All pigs were vital, phenotypically healthy and gained a mean 42% (±19%) weight until slaughter.

### 4.2. TUNEL Assay

The middle parts of the right longissimus dorsi (LD) muscles of siblings with normal and low birth weight, respectively, were snap frozen in liquid nitrogen immediately after sacrifice. Serial transverse 10 μm sections were cut at −20 °C using a cryotome (Leica Biosystems, Wetzlar, Germany). TUNEL assay was performed using the In Situ Cell Death Detection Kit, Fluorescein (Sigma-Aldrich, Darmstadt, Germany) according to the manufacturer’s instructions, and samples were mounted using ROTI Mount FluorCare DAPI (Carl Roth, Karlsruhe, Germany). The number of apoptotic cells, as well as the total cell number, was determined in 10 randomly selected fields for each sample; on average, 10,207 nuclei per sample were analyzed.

### 4.3. Isolation of Cell Subpopulations SPF and SPS

Myogenic cells were isolated from semimembranosus (SM) and LD muscle and cultivated as described previously [55]. Briefly, tissue was minced and digested with Trypsin (4000 U/mL, Sigma Aldrich) before separating cellular subpopulations via Percoll (Sigma Aldrich) gradient centrifugation. Subpopulations were named subpopulation fast (SPF) and subpopulation slow (SPS) according to their proliferative capacity [20,55]. Cell number, size, and viability were determined using Countess Automated Cell Counter (Thermo Fisher Scientific, Darmstadt, Germany).

For proliferation, cells were cultivated for 4 days (passage 0), passaged using 0.05% Trypsin/0.02% EDTA (PAN-Biotech, Aidenbach, Germany), and expanded for another 4 days (passage 1) in proliferation medium (αMEM Eagle (PAN Biotech), 20% fetal bovine serum (FBS, Life Technologies, Darmstadt, Germany), 100 U/mL penicillin/streptomycin (PAN Biotech), 2.5 µg/mL amphotericin (PAN Biotech), and 0.05 mg/mL gentamycin (PAN Biotech)). For flow cytometry analysis, cells were passaged again and cultivated until day 14 after isolation (passage 2).

### 4.4. Isolation of Cells from Single Myofibers

Single, intact myofibers were obtained from fibularis tertius (FT) muscle, as described previously [59]. In short, muscle was digested with 0.2% collagenase type I (Clostridium histolyticum, Merck, Darmstadt, Germany) and 0.1% elastase from porcine pancreas (SERVA, Heidelberg, Germany), and single myofibers were derived via titration. Cells were allowed to migrate from myofibers to cell culture dishes in proliferation medium (DMEM with 4.5 g/L glucose (PAN-Biotech), 20% FBS (Life Technologies), 0.5% chicken embryo extract (Biomol, Hamburg, Germany), 100 U/mL penicillin/streptomycin, and 2.5 μg/mL amphotericin B). Myofibers were removed 2 days after isolation.

### 4.5. Differentiation Assay

Cell subpopulations were seeded on Primaria-treated 24-well plates (VWR, Hannover, Germany) coated with Matrigel (growth factor reduced, Th. Geyer, Renningen, Germany) directly after isolation (for gene expression analysis) or at day 4 after isolation (for immunofluorescence staining). Cells obtained from single myofibers were seeded on Nunc 4-well plates (Life Technologies, Darmstadt, Germany) coated with Matrigel directly after isolation. When reaching approximately 80–90% confluency, serum concentration in medium was reduced to 2% to induce myogenic differentiation.

### 4.6. Flow Cytometry Analysis

Proliferating cell subpopulations were stained against Desmin, MyoG, and MHC at day 4, 8, and 14 after isolation for flow cytometry analysis, as described in [55]. Deviating from that, for MyoG detection, rabbit anti-MyoG antibody (1:100, Biorbyt, Cambridge, UK) was used.

### 4.7. Immunofluorescence Staining of Differentiating Cells

Cell subpopulations, as well as cells obtained from single myofibers, were allowed to differentiate until myotube formation was observed and were subsequently stained against MyoG and MHC simultaneously. Differentiation medium was added to cell subpopulations at day 7 or 9 after isolation (mean: day 7.17), and cells were fixed between day 11 and day 16 after isolation (mean: day 14). Thus, the mean duration of differentiation was 6.83 days. Cells derived from single myofibers were induced to differentiate between day 4 and day 13 after isolation (mean: day 6.9) and fixed between day 10 and day 21 after isolation (mean: day 14.4). The mean duration of differentiation was 7.5 days.

Cells were fixed in 4% paraformaldehyde, washed with PBS, and permeabilized with 0.5% TritonX100 for 20 min, and they were blocked in 0.5% TritonX100 containing 20% rabbit serum for at least 1 h. Mouse anti-MyoG antibody (IgG1, 1:50, Abcam, Berlin, Germany) was diluted in mouse anti-MHC antibody (MF20, supernatant, IgG2b, Developmental Studies Hybridoma Bank; [73]) containing 0.5% TritonX100 and incubated overnight. After washing again with PBS, cells were incubated with the corresponding secondary antibodies (both 1:1000, Thermo Fisher Scientific) goat anti-mouse IgG2b (Alexa Fluor 488, Thermo Fisher Scientific) and goat anti-mouse IgG1 (Alexa Fluor 546, Thermo Fisher Scientific) for 45 min. Cell nuclei were stained using DAPI (1 µg/mL). Images were acquired using Leica DM4000B (Leica Biosystems) with Leica QWin V3 software and merged with Adobe Photoshop CS4. Contrast and brightness were adjusted to the same degree within every sample group. An amount of 5–6 random sections per sample were analyzed using Fiji 1.52n [74,75]. Myotube area (multinucleated MHC+ area) was identified and quantified, and the number of cell nuclei as well as the number of MyoG+ nuclei were determined inside and outside of myotube area. Subsequently, the following parameters could be calculated: fusion rate (percentage of cell nuclei found in myotubes), (mean) myotube size, myotube number (number of myotubes per 1000 nuclei), myotube nuclei (mean number of nuclei per myotube), and percentage of MyoG+ cells.

### 4.8. Migration Assay

After 7–8 days of isolation, cell subpopulations or fiber-derived cells were seeded in 4-well µ-slides (ibiTreat surface, ibidi, Gräfelfing, Germany) in a 2-well culture insert (ibidi) to perform exclusion zone assay. After reaching confluency, cells were incubated with 10 µg/mL Mitomycin C (Abcam) for 2 h at 37 °C to inhibit proliferation [61]. Subsequent removal of the insert created a 500 µm cell free gap. In contrast to the scratch or wound healing assay, this assay avoids physical damage of cells, as a defined area is physically blocked and cannot be covered by cells until the barrier is removed. Directly after taking out the insert and after 19–20 h (cell subpopulations) or 14 h (cells obtained from myofibers), pictures showing the gap were taken at three fixed positions (Primovert, Zeiss, Oberkochen, Germany) to quantify the width of the (remaining) gap in order to calculate the percentage of gap closure ((distance of original gap − distance of remaining gap)/distance of original gap × 100%; [76]).

### 4.9. Gene Expression Analyses

Cell subpopulations were seeded for differentiation assay directly after isolation, induced before reaching confluency (between day 2 and day 9; mean: day 3.96), and collected when inducing differentiation (d0) and 2, 4, and 6 days later.

Isolation of total RNA and cDNA synthesis was performed using NucleoSpin RNA Plus XS with DNA removal column (Machery & Nagel, Düren, Germany) and iScript cDNA Synthesis Kit (BioRad, Feldkirchen, Germany) according to the manufacturer’s instructions. Samples were run in duplicates using a final reaction volume of 20 µL (10 µL Vazyme AceQ qPCR SYBR Green Master Mix (Absource Diagnostics, Munich, Germany), primers (0.6 µL of 0.3 µM each), and 12.5 ng cDNA). The PCR program (95 °C 600 s, 45 cycles of 95 °C 20 s, 60 °C 30 s, 72 °C 20 s) and subsequent melting curve analysis (95 °C 10 s, 65 °C 60 s, 97 °C 1 s) were carried out in the Light Cycler 96 System (Roche, Mannheim, Germany). For detection of *MyH1*, *MyH2*, and *MyH4*, the following program was used: 95 °C 180 s, 45 cycles of 95 °C 15 s and 60 °C 90 s [20]. Samples without cDNA served as a negative control for each set of primers. Cq values were calculated and normalized against the reference genes *Ppia*, *Csnk2A2*, and *Yhwaz* before calculating relative gene expression using the E-ΔΔCq method [77], with E being the sample-specific primer efficiency determined by LinRegPCR (Version 2020.0) [78,79,80]. The E-ΔΔCq values of each sample were normalized against the mean value of SPF cells from NBW piglets at day 0 for the respective target gene. Primer sequences can be found in Table 1.

### 4.10. Statistical Analyses

Data are presented as mean ± SD. For statistical analysis, SigmaPlot 13.0 (Systat Software GmbH, Frankfurt am Main, Germany) was used to compare values of NBW and LBW piglets. If Normality Test (Shapiro–Wilk) and Equal Variance Test (Brown–Forsythe) were passed, a one-tailed students *t*-test was performed. Otherwise, the Mann–Whitney Rank Sum Test was performed. A *p*-value of ≤0.05 was considered statistically significant. For analyzing myotube size after differentiation assay, myotubes were assigned into three groups according to quartiles of frequency distribution (25% small, 50% middle, and 25% large) using IBM SPSS Statistics 22.

## 5. Conclusions

The reduced bioavailability of MPC in LBW piglets, the shifted proportion of cellular subpopulations at the expense of fast proliferating cells, and the reduced proliferation rate of these cells contribute to a worsened status quo in LBW piglets for muscle development and growth. Although the onset of the differentiation process seems to be not affected in LBW piglets, our investigation clearly shows that, in muscles from LBW piglets, two important factors for robust myofiber hypertrophy, namely provision of myonuclei and fusion rate, were disturbed. Notably, obtaining cells with different methods and the detailed (although time-consuming) analyses of distinct quantitative parameters are worthwhile to gain deeper insights into pathophysiological mechanisms.

## Figures and Tables

**Table 1:** Primer sequences.

Target Gene	Forward (5′-3′)	Reverse(5′-3′)
*Pax7*	gacccctgcccaaccacatc	acatccggagtcgccacct
*Myf5*	tgtaccaaatgtatatgccacggat	atcggtgctggcaactggag
*MyoD1*	actgttccgacggcatgatgga	gctcgacaccgcagcattctt
*MyoG*	cgcagcgccatccagtacat	gcagatgatcccctgggttgg
*Desmin*	tttgctagtgaggccagcgg	ggatagggaggttgatccggc
*Mrc1*	tggagcagatggaaggtttatgg	acttgaatggaaacgcacagg
*MyoF*	gggagaatttaagattgatgtcgg	ctggtatcttctgggtcattgag
*Mymk*	tttctgcgccttgacatcct	acgccaaacatcacgaaagtc
*MyH1* [81]	gggtctacgcaaacacgagaga	cagatcctggagcctgagaatg
*MyH2* [82]	gcaaaagcgtaatgctgaagct	cctcttccgtctggtaggtgagt
*MyH3* [81]	tgtccaaggcgaaggccaac	ggtcagctcgctcatgctcc
*MyH4* [81]	cactttaagtagttgtctgccttgag	ggcagcagggcactagatggt
*Ppia*	cgcgtctccttcgagctgttt	gaagtcaccaccctggcacat
*Csnk2A2*	agtctcacgtcccgagctg	tgttccaccacgaaggttctcc
*Yhwaz*	gaactccccagagaaagcctgc	gggtatccgatgtccacaatgtc

## Data Availability

The data presented in this study are available on request from the corresponding author.

## Abbreviations

The following abbreviations are used in this manuscript:

FBS	Fetal bovine serum
FT	Fibularis tertius
LBW	Low birth weight
LD	Longissimus dorsi
MHC	Myosin heavy chain
MMC	Mitomycin C
MPC	Myogenic progenitor cells
Mrc1	Mannose receptor 1
MyoF	Myoferlin
MyoG	Myogenin
Mymk	Myomaker
NBW	Normal birth weight
SC	Satellite cells
SM	Semimembranosus
SPF	Subpopulation fast
SPS	Subpopulation slow
